# Implant insertion angle and depth: Peri-implant bone stress analysis by the finite element method

**DOI:** 10.4317/jced.58930

**Published:** 2021-12-01

**Authors:** Fabiano Rito-Macedo, Millena Barroso-Oliveira, Luiz-Renato Paranhos, Joelson Rodrigues-Brum, Igor-Felipe Pereira-Lima, Fabiana-Mantovani Gomes-França, Rui-Barbosa de Brito-Junior

**Affiliations:** 1 Assistant Professor of Periodontics, State University of Amazonas (UEA), Manaus, Amazonas, Brazil; 2 Post-Graduation Program in Dentistry, Federal University of Uberlândia (UFU), Uberlândia, MG, Brazil; 3 Division of Preventive and Community Dentistry, School of Dentistry dentists, Federal University of Uberlândia (UFU), Uberlândia, MG, Brazil; 4 Assistant Professor of Endodontics, State University of Amazonas (UEA), Manaus, Amazonas, Brazil; 5 Department of Oral Pathology, Federal University of Rio Grande do Sul (UFRGS), Porto Alegre, Rio Grande do Sul, Brazil; 6 Associate Professor, Faculdade São Leopoldo Mandic, Campinas, São Paulo, Brazil

## Abstract

**Background:**

The study aimed to assess the influence of different implant insertion angles and depths on the stresses produced on the surface of peri-implant bone tissue under axial and oblique loading.

**Material and Methods:**

The entire study followed the recommendations of the Checklist for Reporting *In-vitro* Studies (CRIS). The implant was placed in the region of element 36, according to the following models: M1 (0 mm / 0°); M2 (0 mm / 17°); M3 (0 mm / 30°); M4 (2 mm / 0°); M5 (2 mm / 17°); M6 (2 mm / 30°). The models were subjected to loading, with intensity of 100 N. The stress assessment followed the Mohr-Coulomb criterion and qualitative and quantitative analyses were performed.

**Results:**

Angled implants and installed below the bone crest produced the highest stresses on the cortical bone, and the axial load presented the highest stress peaks on the buccal side of implants perpendicular to the bone crest. Regardless of the type of load (axial or oblique), inclined implants presented the highest stress peaks on the lingual side of the cortical bone.

**Conclusions:**

Implants installed perpendicular to and with a prosthetic platform at bone crest height provided the lowest stresses to peri-implant bone tissue under both axial and oblique loading.

** Key words:**Finite element analysis, dental implants, axial loading, biomechanical phenomena.

## Introduction

The current advances in implantology, since the advent of osseointegration, have been propelling the development and production of new dental implant systems with state-of-the-art design, surface treatments, and materials in favor of offering treatments with the most predictable, comfortable, and accessible implant-supported prostheses to all patients (1).

Along with the development of new implant systems, there was an increased need for investigation and research in the field to understand the actual effects of such modernism in the behavior of peri-implant tissues (2,3). Hence, studies assessing bone behavior in the treatment with dental implants have been showing that among the main enemies of peri-implant tissues are the absence of planning and implant placement in sites and positions not recommended by the manufacturers (4).

Factors such as the intensity and direction of forces received (axial or oblique) associated with the degree of implant insertion angle to the bone crest may affect directly the stability of the implant-prosthesis system (4), as well as the total of stresses produced on the surface of peri-implant bone tissue (5-7). Moreover, the levels of stress exerted in the bone tissue were also affected by bone quality and implant insertion depth (8).

Based on this, finite element analysis has played an important role in accurately analyzing the magnitude and distribution of the mechanical stress around implants, representing a valuable strategy to study the macroscopic morphology of implants and peri-implant tissues (9). Therefore, considering the lack of information regarding bone behavior in the installation of Straumann™ Bone Level Tapered (BLT) implant systems at different insertion angles and depths, the present study aimed to investigate, with finite element analysis, the influence of these insertion angles and depths on the stress produced on the surface of peri-implant bone tissue under axial and oblique loading.

## Material and Methods

The study was submitted to the ethics committee and dismissed from analysis, according to protocol #2017/0745. The entire study was developed according to the recommendations of the Checklist for Reporting *In-vitro* Studies (CRIS) (10).

- Obtaining the models

The mandibular geometric model was obtained online from a model available for the free use of the scientific community. The geometric changes required were performed in the CAD Solidworks 2017 software (Dassault Systemes, Solidworks Corps, USA). To shape the external geometry of the future implant prosthesis, the models were edited and the edentulous mandible was joined to the model of tooth 36 of the dentate mandible.

Some adjustments were required in the mandibular model. The thickness of the cortical bone was increased by 0.5 mm, turning 2 mm into 2.5 mm, so that the entire simulated implant maintained the anchorage of the platform in the cortical bone. Moreover, because the models were composed of inclined intermediates, with a minimum height of 2.5 mm available from the manufacturer, a bone loss located in the posterior region of 2 mm was modeled, aiming to allow a suitable future crown in all models.

To obtain the geometric models of the implant and the components used in the study, they were subjected to reverse engineering with a digital caliper (Mod. 500-196-30B, Mitutoyo Sul Americana Ltda., Suzano, Brazil), digital microscope (MV500UM-PL, Cosview Technologies Co. Ltd, Bantian, China) with a magnification of 5x ~200x, and a measuring software (Miviewcap 6.0, Cosview Technologies Co. Ltd, Bantian, China) to measure the geometry of the components and allow their modeling in the Solidworks software.

- Sample preparation

**Figure 1:**
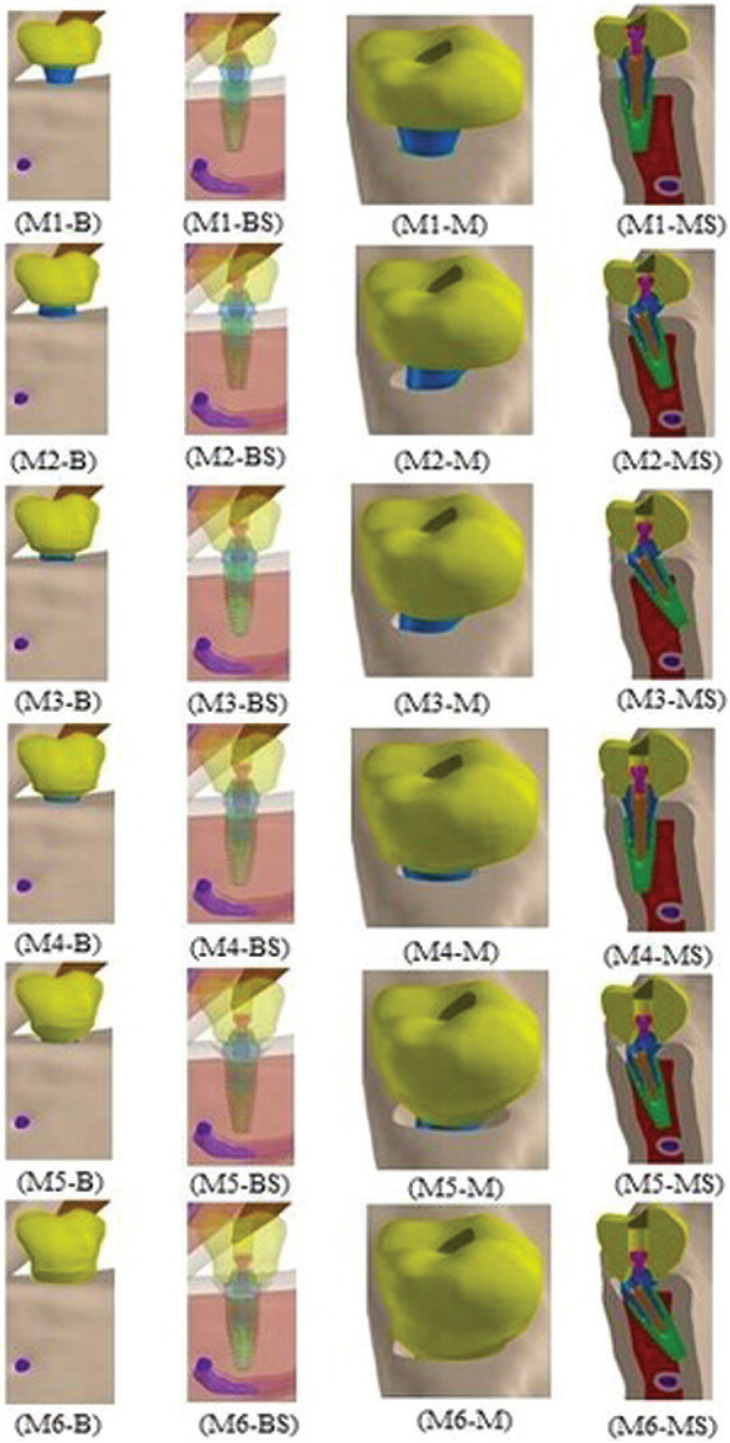
Models analyzed. Buccal (B), buccal semitransparent (BS), mesial (M), and mesial sectional (MS) views.

The Straumann™ Bone Level Tapered (BLT) Cone Morse (CM) implant (4.1 x 10 mm, Institut Straumann AG, Basel, Switzerland), with an intermediate of Ti-6Al-7Nb alloy, RC abutment (2.5 mm, Institut Straumann AG, Basel, Switzerland), was placed in the region of element 36 according to the following models: M1 or control - implant perpendicular to the bone crest with a platform at crest height and straight intermediate; M2 - implant angled at 17° relative to the bone crest with a platform at crest height and intermediate angled at 17°; M3 - implant angled at 30° relative to the bone crest with a platform at crest height and intermediate angled at 30°; M4 - implant perpendicular to the bone crest with platform 2 mm below the crest and straight intermediate; M5 - implant angled at 17° relative to the bone crest with platform 2 mm below the crest and intermediate angled at 17°; M6 - implant angled at 30° relative to the bone crest with platform 2 mm below the crest and intermediate angled at 30°. The reference of the bone crest was based on the buccal edge of the implant platform. Figure 1 shows the different models analyzed in the study.

- Determination of contact points

The crown of the models was made of lithium disilicate glass-ceramics with a minimum thickness of 1.5 mm (IPS e.max press, Ivoclar, Vivadent, Schaan, Liechtenstein). The structure that simulated the occlusal third of antagonist teeth was made of enamel and received contact points for the application of axial and oblique loads. For the axial load, three round contact points with 1 mm of diameter were placed: two in the buccal cusp on the buccal and lingual sides and one in the lingual cusp. For the axial load, a bolus with an approximate thickness of 2 to 3 mm was placed between the crown and the antagonist structure. In turn, for applying the oblique load, the points were positioned in the buccal sides of the buccal cusps.

- Finite element analysis

For the finite element analysis, all models were exported to the Ansys Workbench V19.1 software (Ansys Inc., Canonsburg, PA, USA). To correctly represent the mechanical behavior of each component, the different elements of the models were set with a modulus of elasticity and Poisson’s coefficient retrieved from the literature, as described in Table 1. All materials were considered isotropic, homogeneous, and linearly elastic. Regarding the mechanical properties of the implant alloy (Roxolid™), due to the absence of reliable tests for the modulus of elasticity of the material, this study used the mean between the modulus of elasticity of a titanium alloy with 10% zirconia and 90% titanium and another with 20% zirconia and 80% titanium (15). For Poisson’s coefficient, the same modulus of titanium grade V was used (18) because the materials have similar properties.

**Table 1:**
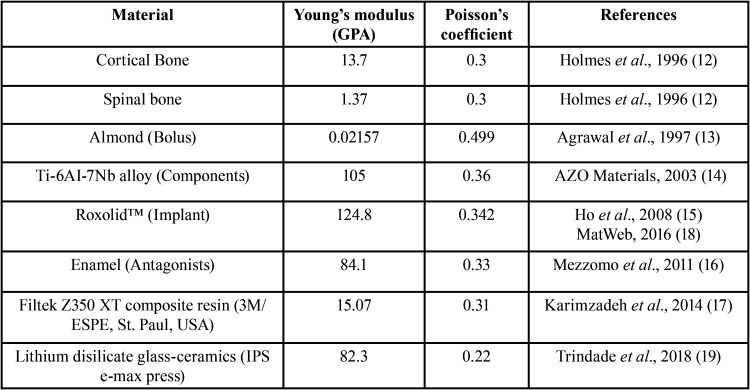
Mechanical properties of the materials.

Non-linear frictional contacts with a friction coefficient of 0.2 μ (20) were simulated for the contact between titanium surfaces. The same value was conveniently used for the contact between the titanium surfaces and the framework. All the other contacts were simulated as sliding contacts or gap formation. The implant was considered osseointegrated.

The models were simulated in two steps. First, pre-stress was applied to the screws. For better standardizing the stresses, a specific resource of the Ansys finite elements software - “bolt pretension” - was used, allowing the application of pre-torque stress with a predetermined force or screw length adjustment. Thus, the mesh refinement was verified with a temporary pre-torque in the intermediate. After adjusting the mesh, the load in the screw was adjusted until representing a peak value of 65% of the limit of proportionality of titanium, by the von Mises equivalent stress criterion, with a tolerance interval of 1%. The limit of proportionality used was 900 MPa for the Ti-6Al-7Nb alloy (14).

The next step consisted of the application of masticatory loads, simulated with 100 N of intensity in the axial and oblique loading patterns. The first pattern, or axial load, was applied with a parallel vector along the axis of the element, in the upper portion of the structure that simulated the antagonist teeth. To simulate the occlusal contact, the antagonist structure was set with frictionless supports on the sides to allow a uniquely gingival occlusal movement. The second pattern, or oblique load, was simulated with a vector toward the buccolingual aspect, forming a 30° angle with the occlusal plane. The antagonist structure was used to standardize the loading area. Rigid supports were added to the areas of masticatory muscles. The simulations were non-linear to the contact.

**Figure 2:**
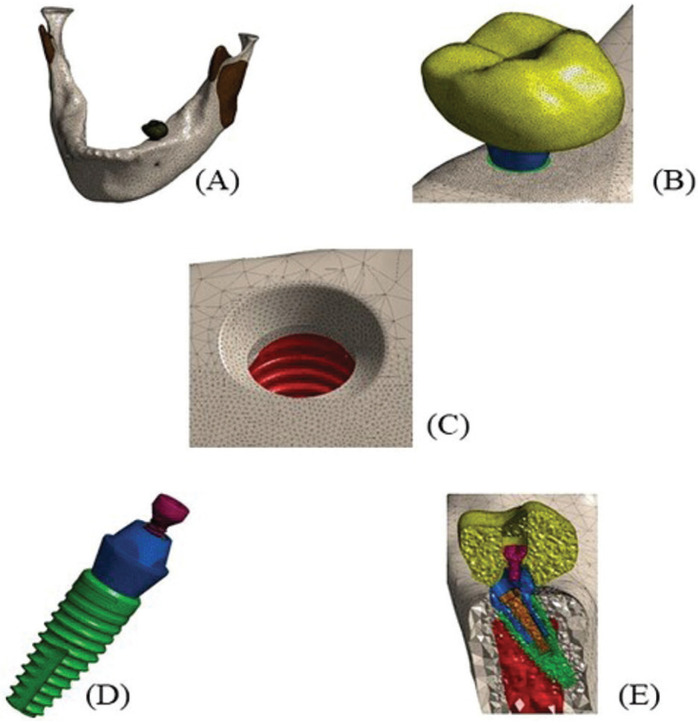
Examples of meshes created. Mandible (A), crown (B), bone structure (C), implant and abutment (D), and all structures in a sectional mesial view (E).

The finite element meshes were then created with a refinement process (≤ 5%) and produced with 10-knot quadratic tetrahedral elements (solid 187), allowing to copy the irregular geometry of the models analyzed. The number of knots/elements ranged from 1087369/667132 to 1378657/854379. Figure 2 shows the meshes created. All models were resolved (Windows 10 64 bits, Intel I7 6800k processor, 112 Gb RAM) and the graphic and numerical plots of the data were registered, assessed, and compared by qualitative and quantitative analyses.

- Assessment of stresses in peri-implant bone tissue

**Figure 3:**
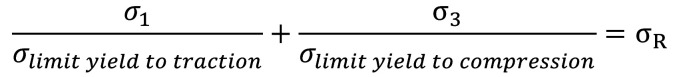
Formula.

The Mohr-Coulomb criterion was used to quantify in a structural level the risk of damage to peri-implant bone tissue. Therefore, to facilitate the comparative analyses, an adaptation from the original formula was used, as follows: (Fig. 3).

where σR is equivalent to the result, σ1 is the main maximum stress, σ3 is the main minimum stress, and σlimit represents the maximum yield stress to compression and traction.

As a reference for the calculation, the limit yield stress to traction was 82.8 MPa and the limit yield stress to compression was 133.6 MPa (21).

## Results

- Stresses on peri-implant bone tissue

The stress assessment values for peri-implant bone tissue according to the Mohr-Coulomb criterion and their percentage relative to control for the axial load were: M1 or control – 0.247 (100%); M2 – 0.219 (88%); M3 – 0.323 (131%); M4 – 0.339 (137%); M5 – 0.472 (191%); M6 – 0.426 (172%). The results for the oblique load were: M1 or control – 0.274 (100%); M2 – 0.352 (128%); M3 – 0.564 (206%); M4 – 0.402 (146%); M5 - 1.304 (476%); M6 – 0.903 (329%).

- Axial load

**Figure 4:**
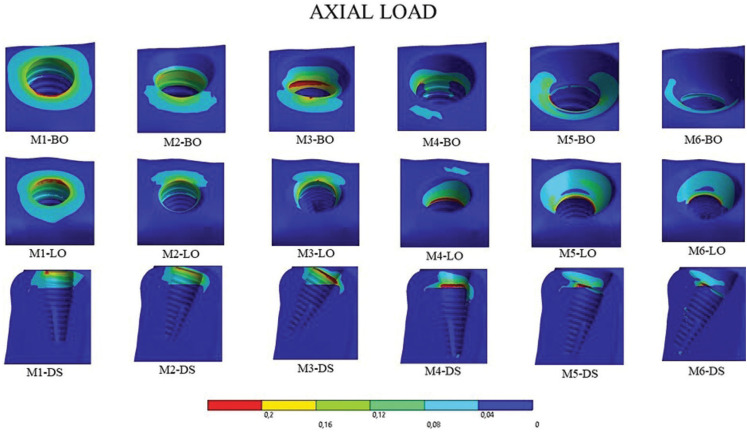
External and sectional view of results on the peri-implant bone under axial loading. BO: buccal occlusal, LO: lingual occlusal, and DS: distal in sectional view. Because it is a sectional view, the distal view shows the mesial portion.

When analyzing the results of the peri-implant bone under axial loading qualitatively, the peaks occurred in the cavosurface region only in the cortical bone. Figure 3 shows, in a sectional distal view, that these peaks occurred on the buccal side of models M1, M2, and M4 and on the lingual side of the cortical bone in models M3, M5, and M6. Quantitatively, the models with perpendicular implants and installed at bone crest height presented the best results (M1 and M4) (Fig. 4).

- Oblique load

**Figure 5:**
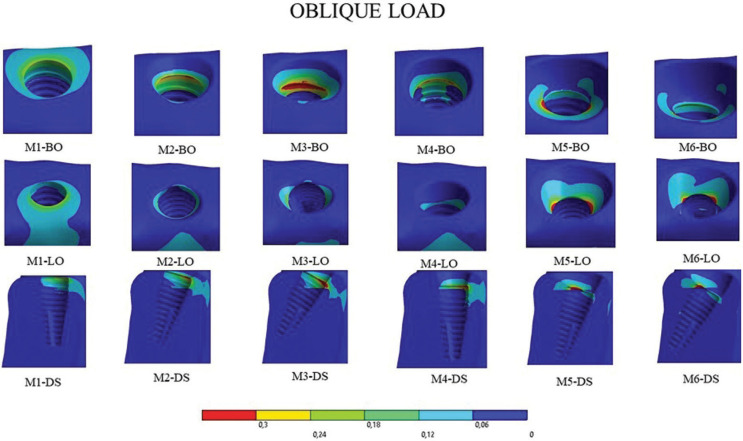
External and sectional view of results on the peri-implant bone under oblique loading. BO: buccal occlusal, LO: lingual occlusal, and DS: distal in sectional view. Because it is a sectional view, the distal view shows the mesial portion.

Under oblique loading, all models presented peaks in the lingual cortical region. Quantitatively, the results were superior to the axial load. Overall, the implant models installed perpendicular to the crest (M1 and M4) presented the best results regarding stress on peri-implant bone tissue (Fig. 5).

## Discussion

The study investigated the influence of different implant insertion angles and depths on the stress levels distributed to peri-implant bone tissues. Implants and prosthetic intermediates angled at 17° and 30° and straight were simulated. The implants were placed at the bone crest level and 2 mm below it. Six models were created, with M1 (0° / 0 mm) as control, and the implant used was the Straumann™ Bone Level Tapered (BLT) implant (22).

Considering the results of qualitative analyses, under axial loading, the stress peaks occurred on the buccal side in models M1, M2, and M4, in a sectional distal view. This is because the bolus fell over the grinding sides, which in lower molars are positioned more to the buccal aspect. However, in the implants of models M3, M5, and M6, the stresses moved to the lingual side of the cortical bone because, due to the angle of implants, the lingual portion tends to present a smaller cortical bone thickness, making it more susceptible to stress concentrations. Results from previous studies show stress on peri-implant bone tissue in the buccal and mesiobuccal aspects around the neck of the implant under vertical loading (23). Under lateral loading, the highest stress was observed in the lingual aspect with a compressive micro-deformation in the cervical margin of the alveolar bone crest, and it may play a critical role in maintaining the bone levels involving the implant (24).

Regardless of the type of load (axial or oblique), implants with bone insertion below the bone crest and at a 30° angle (M6) presented better results than implants with insertion of 17° to the bone crest (M5). This is due to the presence of a larger contact area in the cortical bone, which is obtained with a higher angle. The stress concentration in the cortical bone around the implant dissipates in the adjacent bone tissue, playing a major role in supporting the functional pressure exerted by the implant (25) due to the elastic modulus of the cortical bone, resulting in higher resistance to deformation (24). Thus, implant stability is directly related to cortical bone thickness (26).

The analysis with the finite element method allowed verifying that implant models installed perpendicular to the bone crest caused the lowest stresses to peri-implant bone tissue. Installing implants in inadequate positions may lead to damages to peri-implant bone tissue. Thus, the literature shows that, in angled models subjected to axial or oblique loads, the stability of the implant/prosthesis system was compromised (4).

Frost (27) proposed the mechanostat theory, in which depending on the deformation applied, the bone may suffer disuse atrophy, maintain its bone mass on physiological loads, increase bone mass with higher stimuli than physiological ones, or suffer resorption when the deformation surpasses the tolerable limit of the organism. Although the theory is extensively discussed regarding the stimuli that determine bone response, the concept of bone mass loss, maintenance, or gain is well accepted by the scientific community, depending on the intensity of the stimulus. Considering the intensity of stresses produced to the bone tissue by the angled implant models in the present study, the risk of bone loss is suggested, especially under oblique loading.

Based on implant insertion depth, the results showed that the implants installed with the prosthetic platform at bone crest height produced lower stresses to the bone tissue than implants placed 2 mm below it. This is because the implant used (Straumann™ Bone Level Tapered (BLT)) is originally for bone level and due to the great thickness of the peri-implant cortical bone of the mandibular model studied. The stress value is usually higher in the cortical bone than the cancellous bone (28,29) but the properties of the cortical bone supported a higher amount of stress. A bone tissue with higher intensity will allow a better stabilization of the set of implant and bone tissue than the bone with lower intensity (29). Thus, the thickness of the cortical bone affects directly the stability and stress reduction between implants and cortical bone tissue (26). The data corroborate the results obtained by Rismanchian *et al*. (30), in which the stress caused to the bone tissue was also higher in implant models placed below the bone crest.

Based on the present study, it was verified that implants installed at the bone level have more contact with the cortical bone tissue, obtaining primary stability that favors an immediate load placement. This result is important from the clinical standpoint, considering there is a possibility to prevent more advanced surgeries (osteogenic distraction and lower alveolar nerve laterality) in regions with short mandibular bone tissue height. In cases requiring a larger prosthetic area for esthetic purposes, installing a buried implant regardless of the angle can represent a choice of surgical technique. However, further clinical studies are not discarded, aiming to bring results as close as possible to the behavior found in living tissues.

The main limitation of the study is the inability of the finite element analysis model to simulate all the characteristics of living tissue. However, a bolus was simulated between the ceramic crown and the antagonist structure in enamel, allowing a satisfactory simulation and closer to the clinical reality of the models studied. Therefore, it is evident the significance of the finite element method as a key point to a coherent clinical indication and predictability.

## Conclusions

The finite element analysis allowed concluding that models with perpendicular implants and installed at bone crest height provided lower stresses to peri-implant bone tissue than angled implants and with a prosthetic platform 2 mm below the crest, under both axial and oblique loading.
